# Spatiotemporal Parameters of 100-m Sprint in Different Levels of Sprinters with Unilateral Transtibial Amputation

**DOI:** 10.1371/journal.pone.0163712

**Published:** 2016-10-04

**Authors:** Hiroaki Hobara, Satoru Hashizume, Yoshiyuki Kobayashi, Masaaki Mochmaru

**Affiliations:** Human Informatics Research Institute, National Institute of Advanced Industrial Science and Technology (AIST), Tokyo, Japan; INSEP, FRANCE

## Abstract

The aim of this study was to investigate differences of the spatiotemporal parameters in a 100-m sprint among elite, sub-elite, and non-elite sprinters with a unilateral transtibial amputation. Using publicly available Internet broadcasts, we analyzed 125, 19, and 33 records from 30 elite, 12 sub-elite, and 22 non-elite sprinters, respectively. For each sprinter’s run, the average velocity, step frequency, and step length were calculated using the number of steps in conjunction with the official race time. Average velocity was greatest in elite sprinters (8.71±0.32 m/s), followed by the sub-elite (8.09±0.06 m/s) and non-elite groups (7.72±0.27 m/s). Although there was a significant difference in average step frequency between the three groups, the effect size was small and the relative difference among the three groups was 3.1%. Statistical analysis also revealed that the average step length was longest in elite sprinters, followed by the sub-elite and non-elite groups. These results suggest that the differences in sprint performance between the three groups is mainly due to the average step length rather than step frequency.

## Introduction

Running-specific prostheses (RSPs) with energy storing capabilities have attracted more and more individuals with lower extremity amputations to running as a form of exercise and athletic competition. More recently, RSPs have allowed amputee runners to compete at athletic levels achieved never before [1, 2]. Theoretically, the average velocity during a 100-m sprint is the product of the average step frequency and average step length. Although both parameters are inversely correlated, an increase in one factor will result in an improvement in sprint velocity, as long as the other factor does not undergo a proportionately similar or larger decrease. Because spatiotemporal parameters are modifiable by sprint training sessions [3], identifying factors affecting these parameters of 100-m sprints in unilateral transtibial amputees will provide coaches and practitioners with a basis for better evaluation of sprint performance and aid in the development of more effective training methods for amputee sprinters.

Several studies have compared biomechanical characteristics between able-bodied sprinters and amputee runners using RSPs during maximal and submaximal running [4–7]. The results of these studies are useful to aid in developing ideal coaching and training regimes by identifying the underlying differences between the two groups. On the other hand, despite the fact that examining sprint performance among different levels of sprinters is useful for training-conditioning programs and the design of effective talent development, less research attention has been given to examining differences in biomechanical characteristics in amputee sprinters using RSPs.

In a previous study, Gajer et al. [8] compared sprint performance between faster and slower groups in able-bodied athletes. The authors found that the faster group had a longer stride length during the entire 100-m race than the slower group. Further, Hunter et al. [9] and Weyand et al. [10] also suggested that step length has strong association with the sprint velocity in able-bodied athletes. However, it is unknown if this longer step length of elite group can be observed in amputee sprinters using RSPs.

The aim of this study was to investigate the differences in the spatiotemporal parameters of a 100-m sprint among elite, sub-elite, and non-elite sprinters with a unilateral transtibial amputation. We hypothesized that the differences in sprint performance between the three groups would mainly be due to the average step frequency rather than step length.

## Methods

### Data collection

Since our data set was obtained from publicly-available Internet broadcasts, we did not obtain informed consent. Institutional review board (Environment and Safety Headquarters, Safety Management Division, AIST) approval was obtained prior to the study. We analyzed 177 races of 64 sprinters with lower extremity amputations from publicly available Internet broadcasts. Based on the classification system created by the International Paralympic Committee (IPC; http://www.paralympic.org/), we included the Men’s T44 class (defined as any athlete with lower limb impairment/s that meets the minimum disability criteria for lower limb deficiency, impaired lower limb passive range of motion, impaired lower limb muscle power, or leg length difference). These races included several Paralympics, the IPC Athletics World Championships, and other national- and international-level competitions from 2004 to 2015 (Table 1). Individual races were excluded from the analysis if the athlete did not complete the race or the athlete’s body was not visible throughout the entire race. T44 sprinters who did not use RSPs were also excluded. Analyzing publicly available data from sport competitions for research purposes were performed by Salo et al. [11] for sprint running using 52 able-bodied sprinters, and by Hobara et al. [12] for prosthetic sprinting using 36 able-bodied and 42 amputee sprinters (25 unilateral and 17 bilateral transtibial amputees).

**Table 1:** Summary of the competitions analyzed. EL, SEL and NEL indicates elite-, sub-elite and non-elite group, respectively.

		Number of Subjects		
Year	Competitions	EL	SEL	NEL
2015	ASEAN Paralympic Games			5
2015	IPC Athletics	11	2	
2015	Parapan 2015	1		
2015	IPC Grandprix London	6		
2015	Mano a Mano Challenge	3		
2015	Jogos Paralimpicos Falta 1 Ano	1		
2015	SEIKO Super Athetics	3	1	1
2015	Shizuoka International	1	1	2
2015	7th Fazza IPC Grand Prix Athletics Competition Dubai	1		
2014	IPC Athletics Grand Prix, Berlin	3		
2014	IPC European Athletics	4		
2014	Meeting De Montreuil	4		
2014	IAAF Diamond League Glasgow 2014	7		
2014	Japan Nationals	1	1	2
2014	Great City Games Manchester	2		
2014	Kumamoto Challenges			1
2014	Kanto Championship	1		
2014	Shizuoka International		3	
2013	IWAS			2
2013	Japan Paralympic			2
2013	Sainsbury's Anniversary Games	6		
2013	Great North City Games	3	1	
2013	T-Meeting Tilburg	1		
2013	IPC Athletics	18		
2013	Shizuoka International	2		1
2012	London DAC	1		1
2012	IDM Leichtathletik German Open Athletics	1		
2012	London Paralympic	11		1
2012	IPC European Championship	4		
2012	Mt. Sac Relays	3		
2012	Occidental Oxy Invite	1		
2012	US National	1		
2012	JPN National	2	2	
2011	Oita Athletics	1	2	
2011	IDM Singen	1		
2011	IWAS			3
2011	US National	2		
2011	Japan Paralympic	1	1	2
2011	Japan Nationals			2
2011	IPC Athletics	5	1	1
2011	Kyushu Challenge Athletics			1
2010	Asia Paralympic		2	2
2009	Manchester BT Paralympic World Cup	1		
2008	Beijing Paralympic	7		
2007	Parapan 2007	1		
2004	Athens Paralympic	3	2	4
	**total**	**125**	**19**	**33**

In the present study, we separated the whole population into three groups based on qualification standards. The elite group (EL) consisted of sprinters who satisfied the A-Qualification Standards of the Men’s T44 class (12.20 s) in the analyzed races (London 2012 Paralympic Games Qualification Guide-Athletics, 2011). The sub-elite group (SEL) consisted of sprinters who could not reach the A-Qualification Standards, but satisfied B-Qualification Standards (12.50 s). The non-elite group (NEL) consisted of sprinters who could not reach the B-Qualification Standards. Consequently, the EL, SEL, and NEL groups consisted of 125 (30 sprinters), 19 (12 sprinters), and 33 (22 sprinters) data, respectively.

### Data analyses

As stated in previous studies [12–14], we determined the average velocity (*V*_100_) of each individual by dividing the official race distance (100 m) by the official race times (*t*_*race*_*)*, which were obtained from each competition’s official website; thus,

$$V_{100}=100/t_{race.}$$ (1)

In the present study, we calculated average step frequency (*f*_step_) as

$$f_{step}=N_{step}/t_{race,}$$ (2)

where *N*_step_ is the number of steps, which was manually counted by the authors. Because *V*_100_ is the product of *f*_step_ and average step length (*L*_step_), we calculated the *L*_step_ by

$$L_{step}=V_{100}/f_{step}.$$ (3)

If we could not count the number of steps, we excluded the data from our analyses. The last step before the finish line was considered to be the last step. If an athlete’s foot was located on the finish line, we considered it as a step [15].

### Statistics

Before the statistical analyses, Levene’s test was performed to ensure that the assumptions of normality and homogeneity of variance were met. Since the assumptions were violated in our data, the Kruskal-Wallis test was used to compare *V*_100_, *f*_step_, and *L*_step_ among the EL, SEL, and NEL groups. We also calculated the effect size (ES) for the Kruskal-Wallis test using Cramer’s *V*. From this effect size calculation, the results were interpreted as small (0.1 to 0.3), medium (0.3 to 0.5), or large (higher than 0.5). If a significant main effect was observed, the Mann-Whitney U test with a Bonferroni correction as post hoc multiple comparison was repeated for all combinations in each variables. Because there were three Mann-Whitney U tests in each variable, the alpha levels were set at 0.016 (0.05/3) and 0.003 (0.01/3). Statistical significance was set at *P* < 0.05. These statistical analyses were executed using SPSS version 19 (IBM SPSS Statistics Version 19, SPSS Inc., Chicago, IL).

## Results

**Fig 1:**
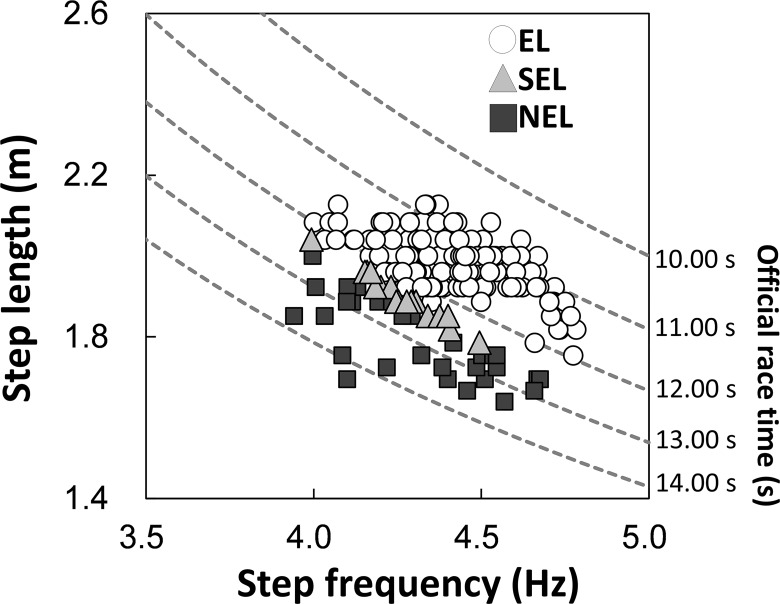
Relationship between *f*_step_ and *L*_step_ for the three groups. Unfilled circles, gray triangles, and filled squares indicate the data for elite (EL), sub-elite (SEL) and non-elite (NEL) groups, respectively. Dotted lines denote the official race times calculated using the combination of *f*_step_ and *L*_step_.

**Fig 2:**
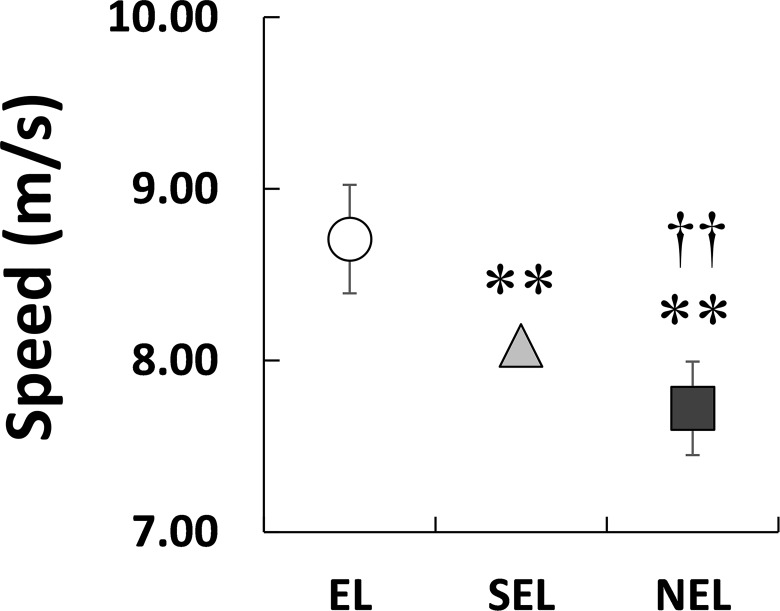
Comparisons of averaged velocity among three groups. Asterisks (**) indicate significant differences with EL at *P* < 0.01. Daggers (††) indicate significant differences with SEL at *P* < 0.01.

**Fig 3:**
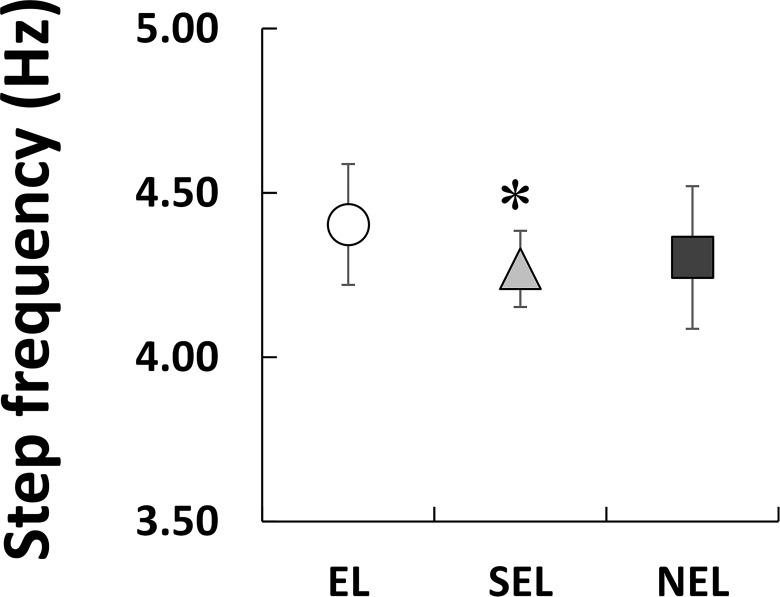
Comparisons of averaged step frequency among three groups. An asterisk (*) indicates a significant difference with EL at *P* < 0.05.

**Fig 4:**
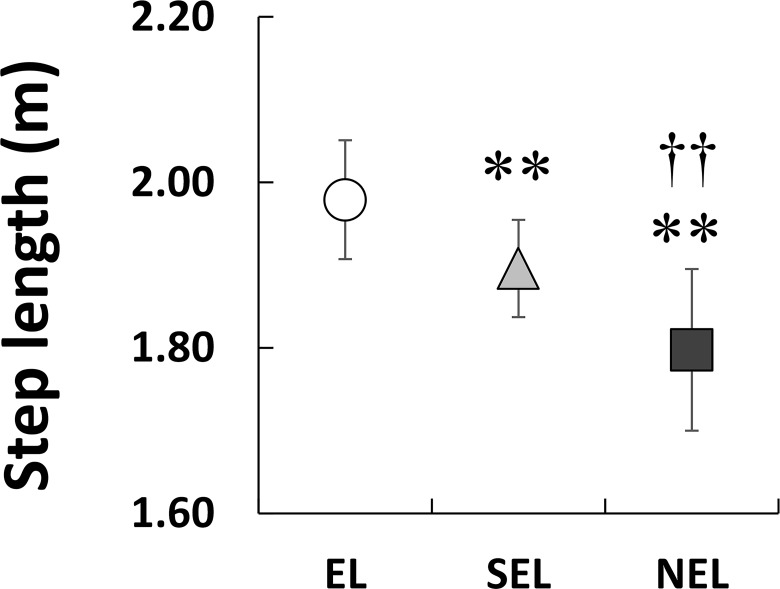
Comparisons of averaged step length among three groups. Asterisks (**) indicate significant differences with EL at *P* < 0.01. Daggers (††) indicate significant differences with SEL at *P* < 0.01.

Fig 1 shows the *f*_step_–*L*_step_ plot for all the individuals in the three groups. Dotted lines indicate the times predicted using the combination of *f*_step_ and *L*_step_. As shown in Fig 2, *V*_100_ exhibited a significant main effect on the groups (*X*^2^(2) = 112.66, *P* < 0.01, ES = 0.57). *V*_100_ was greatest in EL, followed by SEL and NEL (*P* < 0.003 for all comparisons). Statistical analyses revealed that *f*_step_ had a significant effect on the groups (Fig 3; *X*^2^(2) = 13.184, *P* < 0.01), and *f*_step_ in EL was significantly higher than SEL (*P* < 0.016) but not NEL. However, the relative difference between EL and SEL was 3.1%, and the ES of the Kruskal-Wallis test was 0.19 (small). *L*_step_ also displayed a significant effect on the groups (Fig 4; *X*^2^(2) = 72.58, *P* < 0.01, ES = 0.45). Statistical analyses also revealed that *L*_step_ was longest in EL, followed by SEL and NEL (*P* < 0.003 for all comparisons).

## Discussion

The aim of this study was to investigate the differences in the spatiotemporal parameters of a 100-m sprint among elite, sub-elite, and non-elite sprinters with a unilateral transtibial amputation. In the present study, *V*_100_ was greatest in EL sprinters, followed by SEL and NEL (Fig 2). Although a statistically significant difference in *f*_step_ between the three groups was identified (Fig 3), the ES for this effect was small (0.19). On the other hand, *L*_step_ was the longest in EL, followed by SEL and NEL (ES = 0.45, medium; Fig 4). Therefore, the results of the present study support our initial hypothesis that the differences in sprint performance between the three groups would mainly be due to the *L*_step_ rather than the *f*_step_.

In a previous study, Hunter et al. [9] introduced a deterministic model for sprint running, especially for both the *f*_step_ and *L*_step_. Based on the deterministic model, determinants of *f*_step_ and *L*_step_ could be partly explained by the relative horizontal and vertical ground reaction force impulse, segment positions, segment inertial parameters, and air resistance. For the ground reaction forces, Rabita et al. [16] found that elite able-bodied sprinters in their study had a 9.7% greater force production capacity than sub-elite able-bodied sprinters. Furthermore, Fortier et al. [17] and Slawinski et al. [18] reported that elite able-bodied sprinters showed better force production capacity during the sprint start and subsequent steps than sub-elite able-bodied sprinters. Therefore, the differences in *L*_step_ among elite, sub-elite, and non-elite sprinters with a unilateral transtibial amputation in our study may be attributed to force production capacity during sprinting.

There are certain considerations that must be acknowledged when interpreting the results of the current study. First, we assumed the athletes completed a step exactly at the 100-m mark [12–14]. However, a previous study [11] subtracted a distance of 0.55 m and a time of 0.52 s from the calculations of averaged step frequency and step length based on their pilot test. Thus, the reliability and accuracy of the current data should be interpreted carefully. Second, we calculated the average step length using the number of steps taken and the official race time as data. However, Nagahara et al. [19] demonstrated that not all the steps in a 100-m sprint have the same length and frequency, indicating that the average step frequency and step length in the present study may not necessarily be representative of any particular part of the sprint. Thus, continuous changes in spatiotemporal parameters during 100-m sprint in the three groups should be investigated. Third, we only investigated athletes with unilateral transtibial amputations who participated in official competitions, such as the Paralympic Games, IPC Athletics World Championship, IPC European Championship, and other national and international competitions. Thus, caution should be exercised in interpretation and generalization of these findings to other classes, such as transfemoral amputees and bilateral transtibial amputees. Finally, although most of T44 sprinters from 2004 to 2015 generally used Flex-Foot Cheetah® (Össur), Cheetah® Xtreme™ (Össur), or 1E90 Sprinter (Ottobock) RSPs, we did not determine each individual’s RSPs, which might indirectly influence the spatiotemporal parameters during sprinting [20–21]. Consequently, the differences in RSPs used within each of the groups may affect the spatiotemporal parameters of the 100-m sprint. Further research is required on whether and how sprint performance changes among different levels of sprinters with unilateral transtibial amputations.

## Conclusion

In summary, we investigated differences in the spatiotemporal parameters of a 100-m sprint among elite, sub-elite, and non-elite sprinters with a unilateral transtibial amputation. The results of the present study suggest that the differences in sprint performance between the three groups is mainly due to the *L*_step_ rather than the *f*_step_.

## Supporting Information

S1 Appendix The data underlying the findings in this study. (XLSX)

## References

[1] Hobara H, Kobayashi Y, Heldoorn TA, Mochimaru M. The fastest sprinter in 2068 has an artificial limb? Prosth Orth Int. 2015; 39: 519–520. 10.1177/0309364614564026 25630333

[2] Nolan L. Carbon fibre prostheses and running in amputees: a review. Foot Ankle Surg. 2008; 14: 125–129. 10.1016/j.fas.2008.05.007 19083629

[3] Bezodis IN, Salo AIT, Kerwin DG. A longitudinal case study of step characteristics in a world class sprint athlete. In Y.-H. Kwon, J. Shim, J.K. Shim & I.-S. Shin (eds). Proceedings of XXVIth ISBS Conference (Seoul, Korea: ISBS). 2008: 537–540.

[4] Brüggemann GP, Arampatzis A, Emrich F, Potthast W. Biomechanics of double transtibial amputee sprinting using dedicated sprinting prostheses. Sports Tech. 2009; 1: 220–227. 10.1002/jst.63

[5] Hobara H, Baum BS, Kwon HJ, Miller RH, Ogata T, Kim YH et al Amputee locomotion: Spring-like leg behavior and stiffness regulation using running-specific prostheses. J Biomech. 2013; 46: 2483–2489. 10.1016/j.jbiomech.2013.07.009 23953671 PMC3786338

[6] McGowan CP, Grabowski AM, McDermott WJ, Herr HM, Kram R. Leg stiffness of sprinters using running-specific prostheses. J Roy Soc Interf. 2012; 9: 1975–1982. 10.1098/rsif.2011.0877 22337629 PMC3385759

[7] Weyand PG, Bundle MW, McGowan CP, Grabowski A, Brown MB, Kram R et al The fastest runner on artificial legs: different limbs, similar function? J Appl Physiol. 2009; 107: 903–911. 10.1152/japplphysiol.00174.2009 19541739

[8] Gajer B, Thepaut-Mathieu C, Lehenaff D. Evolution of stride and amplitude during course of the 100 m event in athletics. New Stud Athl. 1999; 14: 43–50.

[9] Hunter JP, Marshall RN, McNair PJ. Interaction of step length and step rate during sprint running. Med Sci Sports Exerc. 2004; 36: 261–271. 10.1249/01.MSS.0000113664.15777.53 14767249

[10] Weyand PG, Sternlight DB, Bellizzi MJ, Wright S. Faster top running speeds are achieved with greater ground forces not more rapid leg movements. J Appl Physiol. (1985). 2000; 89: 1991–1999. 11053354 10.1152/jappl.2000.89.5.1991

[11] Salo AI, Bezodis IN, Batterham AM, Kerwin DG. Elite sprinting: are athletes individually step-frequency or step-length reliant? Med Sci Sports Exerc. 2011; 43: 1055–1062. 10.1249/MSS.0b013e318201f6f8 20980924

[12] Hobara H, Kobayashi Y, Mochimaru M. Spatiotemporal variables of able-bodied and amputee sprinters in men’s 100-m sprint. Int J Sports Med. 2015; 36: 494–497. 10.1055/s-0034-1387794 25700099

[13] Hobara H, Potthast W, Müller R, Kobayashi Y, Heldoorn TA, Mochimaru M. Normative spatiotemporal parameters during 100-m sprints in amputee sprinters using running-specific prostheses. J Appl Biomech. 2016; 32: 93–96. 10.1123/jab.2014-0297 26251966

[14] Hobara H, Sano Y, Kobayashi Y, Heldoorn TA, Mochimaru M. Step frequency and step length of 200-m sprint in able-bodied and amputee sprinters. Int J Sports Med. 2016; 37: 165–168. 10.1055/s-0035-1564171 26509370

[15] Hobara H, Hashizume S, Kobayashi Y, Usami Y, Mochimaru M. Ethnicity and spatiotemporal parameters of bilateral and unilateral transtibial amputees in a 100-m sprint. SpringerPlus. 2016; 5: 343 10.1186/s40064-016-1983-1 27066362 PMC4794476

[16] Rabita G, Dorel S, Slawinski J, Sàez-de-Villarreal E, Couturier A, Samozino P et al Sprint mechanics in world-class athletes: a new insight into the limits of human locomotion. Scand J Med Sci Sports 2015; 25: 583–594. 10.1111/sms.12389 25640466

[17] Fortier S, Basset FA, Mbourou GA, Favérial J, Taesdale N. Starting block performace in sprinters: A statistical method for identifying discriminative parameters of the performance and an analysis of the effect of providing feedback over 6-wek period. J Sports Sci Med. 2005; 4: 134–143. 24431969 PMC3880880

[18] Slawinski J, Bonnefoy A, Leveque JM, Ontanon G, Riquet A, Dumas R et al Kinematic and kinetic comparisons of elite and well-trained sprinters during sprint start. J Strength Cond Res. 2010; 24: 896–905. 10.1519/JSC.0b013e3181ad3448 19935105

[19] Nagahara R, Naito H, Morin JB, Zushi K. Association of acceleration with spatiotemporal variables in maximal sprinting. Int J Sports Med. 2014; 35: 755–761. 10.1055/s-0033-1363252 24577864

[20] Baum BS, Schultz MP, Tian A, Shefter B, Wolf EJ, Kwon HJ et al Amputee locomotion: determining the inertial properties of running-specific prostheses. Arch Phys Med Rehab. 2013; 94: 1776–1783. 10.1016/j.apmr.2013.03.010 23542403 PMC3793256

[21] Noroozi S, Rahman AGA, Khoo SY, Zahedi S, Sewell P, Dyer B, et al The dynamic elastic response to impulse synchronisation of composite prosthetic energy storing and returning feet. Proc I Mech E Part P: J Sports Eng Tech. 2014; 228: 24–32. 10.1177/1754337113501491

